# The eCALM Trial-eTherapy for cancer appLying mindfulness: online mindfulness-based cancer recovery program for underserved individuals living with cancer in Alberta: protocol development for a randomized wait-list controlled clinical trial

**DOI:** 10.1186/1472-6882-13-34

**Published:** 2013-02-16

**Authors:** Kristin A Zernicke, Tavis S Campbell, Michael Speca, Kelley McCabe-Ruff, Steven Flowers, Dale A Dirkse, Linda E Carlson

**Affiliations:** 1 Department of Psychology, University of Calgary, Calgary, Alberta, Canada; 2 Department of Psychosocial Resources, Tom Baker Cancer Center, 2202 – 2nd Street SW, Calgary, Alberta, T2S 3C1, Canada; 3 Department of Oncology, University of Calgary, Calgary, Alberta, Canada; 4 eMindful Inc., Vero Beach, Florida, USA; 5 Mindful Living Programs, Chico, California, USA; 6 Enloe Medical Center, Chico, California, USA

**Keywords:** Mindfulness, Cancer, Oncology, Randomized waitlist controlled trial, Online, Stress reduction, Meditation, Yoga

## Abstract

**Background:**

Elevated stress can exacerbate cancer symptom severity, and after completion of primary cancer treatments, many individuals continue to have significant distress. Mindfulness-Based Cancer Recovery (MBCR) is an 8-week group psychosocial intervention consisting of training in mindfulness meditation and yoga designed to mitigate stress, pain, and chronic illness. Efficacy research shows face-to-face (F2F) MBCR programs have positive benefits for cancer patients; however barriers exist that impede participation in F2F groups. While online MBCR groups are available to the public, none have been evaluated. Primary objective: determine whether underserved patients are willing to participate in and complete an online MBCR program. Secondary objectives: determine whether online MBCR will mirror previous efficacy findings from F2F MBCR groups on patient-reported outcomes.

**Method/design:**

The study includes cancer patients in Alberta, exhibiting moderate distress, who do not have access to F2F MBCR. Participants will be randomized to either online MBCR, or waiting for the next available group. An anticipated sample size of 64 participants will complete measures online pre and post treatment or waiting period. Feasibility will be tracked through monitoring numbers eligible and participating through each stage of the protocol.

**Discussion:**

47 have completed/completing the intervention. Data suggest it is possible to conduct a randomized waitlist controlled trial of online MBCR to reach underserved cancer survivors.

**Trial registration:**

Clinical Trials.gov Identifier: NCT01476891

## Background

Receiving a diagnosis of cancer can be highly stressful, requiring psychological and behavioral adjustments to cope effectively with increased levels of stress that subsequently may affect psychological functioning [1] and cancer symptom severity [2]. The prevalence of clinical levels of distress in individuals with cancer is in the 35%–45% range [3-6]. Distress in these individuals most commonly presents as anxiety and mood disorders across stage and site of illness [3,7-9]. Therefore, the development and testing of accessible psychosocial interventions intended to reduce stress and improve mood and quality of life are important [10].

Interest in the potential health benefits of mindfulness meditation within the western medical system has increased with the development and proliferation of interventions modeled after the original Mindfulness-Based Stress Reduction (MBSR) program at the University of Massachusetts Medical Center, developed in the late 1970s by Jon Kabat-Zinn and colleagues [10,11]. Mindfulness meditation is the practice of cultivating moment-to-moment awareness of internal and external experience in an accepting and open manner [12]. MBSR is an 8-week group intervention consisting of intensive training in mindfulness meditation and Hatha yoga that is designed to treat symptoms of stress, pain and chronic illness [12]. Attitudes of open inquiry, patience, suspended judgment and compassion are encouraged and cultivated through the program during class and also through assigned daily homework. Individuals are taught to focus attention on sensations of the breath, body, and objects that enter awareness, such as thoughts and emotions, with the intention to fully experience the present moment [12]. One result of such focused attention in the present moment is reduction of rumination on the past or persistent worry about the future, as well as increased tolerance of uncomfortable emotional experiences (improved emotional regulation), which can result in decreases in symptomatology.

Research indicates F2F MBSR interventions are efficacious for treating a variety of symptoms associated with a range of chronic medical and psychiatric problems, including cancer [13-20]. Current literature, including the body of work from our research team on MBSR in oncology shows participation in F2F MBSR results in decreased stress symptoms, mood disturbance, anger, and fatigue, with concurrent increases in sleep quality, post-traumatic growth, spirituality and enhanced quality of life [14,21-29], as well as changes in several potentially important cancer biomarkers including immune function and stress hormones [30,31]. Several meta-analytic and comprehensive reviews of the effects of F2F MBSR in cancer concluded that it is a clinically valuable evidence-based intervention for cancer patients [16-18,20] with average Cohen’s *d* effect sizes for improving psychological and physical well-being of 0.48 and 0.18, respectively [20].

Despite their proven efficacy, there may be practical and psychological barriers to participation in F2F MBSR programs, such as geographic distance, cancer-related illness, fatigue, limited mobility or disability, child care, transportation, time, and self-consciousness, to name but a few. The Internet represents a promising method of delivering psychosocial interventions such as MBSR to underserved cancer patients who are unable to attend F2F programs. With the increase in Internet use and capabilities, psychosocial interventions are beginning to be offered online [32], and the use of synchronous online therapy that takes place in “real time” has increased. Fast broadband connection allows auditory and video exchanges that simulate the speed of F2F conversations, and videoconferencing using a broad range of software and programs is gaining greater use in therapy contexts [33,34]. In a meta-analysis, Barak et al., in 2008, compared the effectiveness of F2F psychological interventions such as Cognitive Behavioral Therapy (CBT) or Prolonged Exposure (PE) to Internet versions, and overall revealed comparable results [34]. Most similar to the proposed online MBSR study, Gardner-Nix and colleagues evaluated a non-randomized 10-week Mindfulness-Based Chronic Pain Management (MBCPM) intervention for chronic pain patients. The MBCPM program was based on the MBSR program, but additional emphasis was placed on learning to observe emotions associated with pain and general health education [35]. Patients received MBCPM via traditional F2F teaching, via videoconferencing at their local hospital, or were wait-listed. Baseline and post intervention measures showed patients in the F2F and videoconferencing groups achieved similar gains in mental health and pain catastrophizing relative to controls. However, the F2F group obtained significantly higher scores on the physical dimension of quality of life and lower pain intensity ratings than the videoconferencing group [35]. The authors concluded that while Internet interventions show potential for treating chronic pain patients, results may be better in person for some outcomes. While this study did use videoconferencing technology, advancements in sophisticated “real-time” technology since 2008 are significant and have potential to substantially enhance patient interaction and the overall patient experience. Our current study represents an advance over this methodology for several reasons: randomization of our participants, elimination of the need for patients to travel to treatment centres - increasing rural and remote individuals’ access despite geographic location, transportation issues, fatigue etc. We are also able to capitalize on the current technology for improved speed and quality of online communication in our trial.

The positive benefits of MBSR in individuals living with cancer have been well documented by our research team. However, practical and psychosocial barriers may impede participation and access to our popular F2F programs [34,36]. The Internet represents a promising method of delivering empirically supported psychosocial interventions such as MBSR to this underserved cancer population who are otherwise unable to participate, but it has never been evaluated in this context.

In this paper, we describe an ongoing trial to assess the efficacy of an online adaptation of an MBSR intervention for individuals diagnosed with cancer, online MBCR; the first of its kind. Participants are randomized to either an adapted online MBSR condition or a wait-list control condition. This study will help set the direction for future treatment studies to further evaluate online MBSR.

## Objectives

The eCALM Study’s primary objective is to determine feasibility – to examine whether moderately to highly distressed individuals diagnosed with cancer will be willing to participate in online 8-week MBSR groups and complete the intervention. This feasibility objective will objectively be assessed through evaluation of recruitment, retention, attendance, adherence and participant satisfaction. The secondary objective is to examine the efficacy of the online synchronous adaptation of an MBSR intervention for individuals diagnosed with cancer compared to a treatment-as-usual (TAU) wait-list group on a range of previously studied patient-reported outcomes (PROs) including mood, symptoms of stress, post-traumatic growth, spirituality and mindfulness.

### Hypotheses

**1)** Participants will be willing to participate and complete an 8-week online MBSR intervention. Our estimated feasibility outcome proportions of 5% interested, 30% eligible, 85% consent, and 85% complete the intervention will be deemed feasible if within 5% of each target; **2)** Participants in the online MBSR treatment condition will demonstrate greater decreases in symptoms of stress and mood disturbance over the course of the intervention, when compared to participants in the wait-list control condition; **3)** Participants in the online MBSR condition will demonstrate greater increases in mindfulness, spirituality and posttraumatic growth over the course of the intervention, when compared to participants in the wait-list condition.

## Methods

### Study design

**Figure 1:**
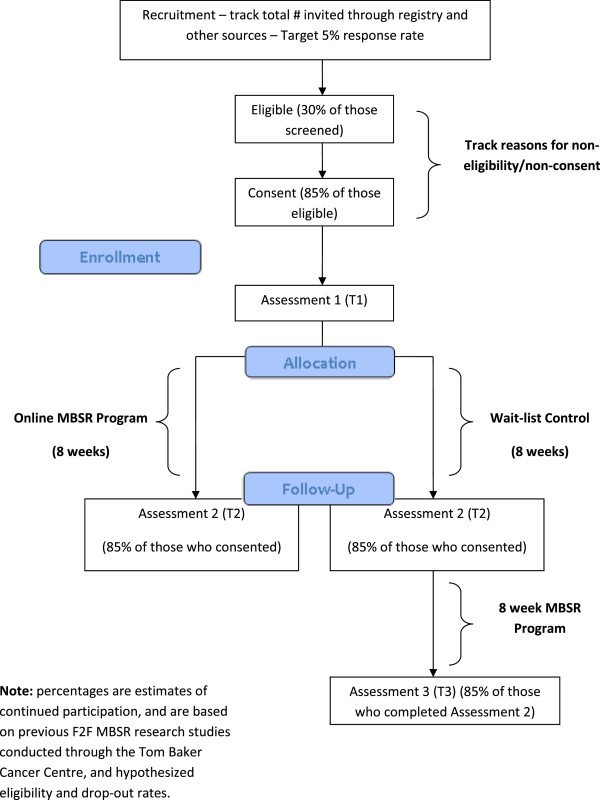
Overall trial design and participant flow diagram.

Figure 1 illustrates the overall study design and participant flow for the trial. This study utilizes a randomized wait-list controlled trial design with pre- and post-assessment. Ethics approval was obtained from the University of Calgary, Conjoint Health Research Ethics Board (CHREB). While the wait-list trial design may limit potential long-term follow-up, preventing conclusions about efficacy of the treatment to maintain effects after this specified time frame, recruitment with a no-treatment control may compromise our ability to accrue participants. Additionally, a wait-list design can control for the influences of pre- and post-treatment assessment, symptom self-monitoring, natural recovery from cancer treatments and spontaneous remission or deterioration of symptoms.

### Eligibility

Inclusion criteria: 1) Age 18 years or older; 2) Speak and read English sufficiently to complete questionnaires; 3) Women and men who have been diagnosed with of any type of cancer, at any time in the past with no restriction on tumour site; 4) Before or currently receiving primary treatment, or completed primary treatment within the last 36 months. This time was chosen in order to assure a group of individuals who were fairly recently involved in diagnosis and treatment, thus also increasing the likelihood that the issues they are dealing with will be more similar, but also including individuals up to 3 years post primary cancer treatment who still feel significant distress; 5) Exhibiting moderate distress as established by responses on the Distress Thermometer (DT) of 4 or greater out of 10. This is an important criterion as statistical problems with floor effects are common in volunteer samples who have relatively high levels of functioning prior to trial involvement; 6) Willing to participate in the intervention requirements; able to participate in the intervention (2 hours per week for 8 weeks, and the full-day retreat), and agree to the randomization procedure; 7) Internet access; self-reported familiarity with computer usage and Internet access with an overall point 5 Megabyte capability and minimum download (2 Megabytes) and upload (500 Kilobytes) is necessary; and 8) Residents in Alberta, who have limited access to existing F2F MBSR programs.

Exclusion criteria: 1) Concurrent self-reported diagnosis of psychosis, bipolar disorder, substance abuse or suicidality. Individuals with depression, anxiety or adjustment disorders are not excluded; 2) Individuals who have previously participated in an F2F MBSR group.

### Recruitment

Potentially eligible participants in Alberta are identified through media outreach, promotional pamphlets, community based networks, and by cancer registry case records to mail study invitation letters. In Alberta, only one major cancer centre has MBSR classes specifically for individuals diagnosed with cancer; therefore all individuals who are unable to attend these F2F classes would be eligible to participate. The Alberta Cancer Registry estimates as of September 2011, 25,000 individuals diagnosed with cancer are eligible to be contacted for the trial. According to research conducted by the PI, approximately 35-45% of these individuals will meet criteria of being distressed. Additionally, 77% of Albertans 16 years of age and older have Internet access and 429 communities throughout the province of Alberta have broadband Internet (http://www.albertacanada.com; http://www.albertasupernet.ca). This forms a potentially large cohort to identify individuals living with cancer who have Internet access for this study.

### Procedures

The study, including its nature, outcomes, and extent of participant involvement, is discussed with all prospective participants. Consent is obtained, and participants are informed that they can refuse to answer any question and they can withdraw from the study at any time. On average the quantitative assessments take 30–45 minutes. Before and after the 8-week online MBSR intervention, participants complete an identical battery of questionnaires (T1 & T2). These assessments are completed online in a secure environment with reminder follow-up emails/phone calls placed to participants who fail to complete their questionnaires within two weeks of expected receipt. Two follow-up email reminders separated one week apart are sent and, if no response, a maximum of three attempts to remind patients via telephone at different times of the day are made, with messages left. Participants who are randomized to the control group complete an additional assessment (T3) following their participation in the MBSR program after waiting.

### Screening, consent and enrollment

At first contact, interested participants are informed of the protocol and randomized design of the study. If interested, researchers ensure that individuals meet basic eligibility criteria. Potentially eligible participants complete the distress screen by phone conducted by the research coordinators. If interested and eligible, study procedures are explained in detail and consent obtained. Consent is also obtained to access medical records to confirm tumour location, stage and treatments received. Due to the lack of F2F contact during screening and consent, additional time is allocated to ensure fully informed consent by providing potential participants the opportunity to ask clarifying questions electronically or telephonically, and to require that the participant affirm that s/he has read the document, understands it, and has been presented with the opportunity to ask questions.

Researchers then provide a questionnaire package (including demographics and medical history, mood, stress, mindfulness, posttraumatic growth and spirituality measures) to complete online. Once a cohort is in place, participants are randomly allocated to one of two conditions using an online research randomization tool. All allocations are computer-based and not predictable. This process eliminates introduction of experimenter bias into participants’ group assignment. Once randomized participants are informed of group assignment (i.e., the immediate MBSR or the wait-list control condition) they are mailed a web-camera, headset, and MBSR course materials before they begin the program. Technical staff guide participants through installation of the equipment and online classroom tools in an orientation session before the first MBSR class. Those participants randomized to the wait-list condition are informed of their program start date and contacted closer to such date to send course materials.

### Masking

The nature of the group assignment and intervention does not allow for masking of participants. Research tasks are assigned to separate members of the team in order to ensure that primary investigators remain blind to participant status, and all questionnaires are completed online, attenuating the influence of bias on the part of research assistants.

### Intervention group – online MBSR for cancer (Mindfulness-based Cancer Recovery – MBCR)

Components of the MBCR program include: didactic instruction, experiential practice, and group process. Didactically, specific topics covered within the 2 hour sessions and in the participant manual are: (a) concepts fundamental to mindful living and mindfulness meditation, (b) emotional, behavioural and cognitive patterns, and how such patterns can influence individual stress responses, and (c) the physical and psychological symptoms of stress and the influence of stress on physical and psychological health. Participants are instructed to apply principles taught didactically, through experiential practice of mindfulness meditation during group sessions and also as homework between formal classes. Guided meditation recordings and videos are distributed to support home practice. Participants are expected to practice 45 minutes of meditation and yoga postures daily. During class sessions the instructor guides participants through experiential activities including gentle Hatha yoga, qigong mindful movement, and various types of mindfulness meditation such as sitting and walking meditations. The instructor facilitates group discussions to encourage conversation around challenges experienced with meditation practice. Both fellow participants and the instructor offer support and feedback to assist in problem solving when difficulties are encountered during meditation practice. The instructor encourages communication and support between group members to enhance the group process [12].

The online MBCR intervention was modeled after the F2F MBCR group intervention described above. The online format that most closely resembles F2F interaction (ability to see, hear and interact with group members in real time) is a synchronous online intervention with video and audio capabilities. In collaboration with the online education company eMindful Inc. (http://www.emindful.com), participants are able to access eMindful’s online virtual classroom and participate in real-time, online, synchronous, 2-hour MBCR classes for 8 consecutive weeks. Participants are asked to engage in the interactive features of the eMindful virtual classroom during the online classes. This includes logging on to the virtual classroom website, watching and listening in “real time” to streaming video and audio of the class instructor and other group participants on their computer screen, “chat” with the instructor and other group participants using a keyboard, and interactively participate using the headsets, web-cameras, and keyboards. Live images through the web-camera facilitate interaction with the instructor and other participants.

The virtual classroom allows for multiple webcams to be viewed by all participants and instructor simultaneously. Online classroom access requires a password and the instructor ensures only approved participants are present in the class. Recorded versions of the classes are available through software that prevents download of the recording (provided through streaming video) as well as password access. This classroom feature allows the PI to monitor MBCR treatment fidelity throughout the trial. Class content and schedule follow the adapted F2F MBCR manual utilized by both PI and Co-I (Drs. Carlson and Speca respectively) and course program published elsewhere [37]. Participants are able to access the manual online or receive a hard copy from research coordinators through post if preferred. The guided meditation recordings and videos are available for participants to download electronically. Participants are provided both orientation/instructions for set up of the new equipment and also an orientation session before the first class where headsets, webcameras, website and classroom functionality are tested and reviewed. This orientation session is completed before the start of the first MBCR class. Each participant is emailed a summary of dates and orientation material before the start of session one. Technical support is continuously provided by eMindful during all online sessions to address any technical issues with the hardware and software, while study related questions are directed to the research coordinators.

### Treatment as Usual (TAU) wait-list control condition

In parallel with the online MBCR program, the TAU wait-list condition completes pre (T1) and post (T2) assessment measures before and after their wait period. Additionally, following the wait period, the control group completes the online MBCR intervention and completes one additional assessment time point (T3).

### Outcome measures and questionnaires

Table 1 contains the outcome measures and their administration timing for the online MBCR and wait-list group participants. Medical history (i.e., type of cancer, dates of diagnosis and treatment, and types of treatment received) and demographic information (i.e., sex, age, ethnic background, education, marital status, and current employment status) is obtained at the first assessment and later verified in the participant’s medical chart, with consent. Participants are also asked to indicate how much experience with meditation and/or yoga they have had previously.

**Table 1:** Outcome measures for the online mbcr and wait-list group participants

***Construct***	***Measure***	***Administration***
Primary Objective: Feasibility	See Figure 1 for estimates –study deemed feasible if within 5% of each target value	
Program Satisfaction	eCALM –Online MBCR Program Evaluation Form	· Post intervention only
Dose of intervention delivered and dose received	Delivered: Attendance log Received: Meditation Log	· Throughout intervention
Distress Screening	Score of 4 or higher on the Distress Thermometer (DT)	· Pre intervention only
Primary Measure of Secondary Objective: Mood	Profile of Mood States (POMS)	· Pre and Post MBCR or wait
Other Secondary Outcomes: Stress	Calgary Symptoms of Stress Inventory (C-SOSI)	· Pre and Post MBCR or wait
Post-Traumatic Growth	Post-Traumatic Growth Inventory-Revised (PTGI-R)	· Pre and Post MBCR or wait
Mindfulness	Five Facet Mindfulness Questionnaire (FFMQ)	· Pre and Post MBCR or wait
Spiritualty	Functional Assessment of Chronic Illness Therapy-Spiritual Well-Being (FACIT-sp)	· Pre and Post MBCR or wait

### Meditation Log and attendance

Information on minutes spent in home practice of meditation and yoga is collected from each participant each week and returned to the study coordinator, which is shared with the group instructor. As part of the online virtual classroom capabilities, a basic feature of conference archiving is utilized. This feature can track all the MBCR classes with a list of attendees, and what time the participants entered and exited the classroom for attendance tracking.

### Screening measures

#### Distress thermometer

The Distress Thermometer (DT) is a 0 to 10 single visual analogue scale oriented as a vertical thermometer [38]. The DT has been validated against clinical diagnosis of anxiety and mood disorders, the Brief Symptom Inventory (BSI), the Hospital Anxiety Depression Scale (HADS), and endorsed for use by the National Comprehensive Cancer Network (NCCN). A cut-off score ≥ 4 has been identified in the literature as optimal with regards to both sensitivity and specificity for labeling individuals diagnosed with cancer as having significant psychological distress [39,40].

### Primary outcome measure

#### Profile of Mood States (POMS)

The POMS is an instrument with 65 items that assesses six affective dimensions and produces a Total Mood Disturbance (TMD) score [41]. Psychosocial interventions (including psychological adaptation to diagnosis and treatment of cancer) frequently use this scale. This instrument measures state (vs. trait) attributes, therefore the POMS scale is an appropriate instrument for repeated measures as previous administrations do not influence later administrations. The Kuder-Richardson overall internal consistency measure of the six subscales ranged from 0.84 (Confusion) to 0.95 (Depression) in two separate studies, with test retest stability ranging from 0.65 (vigor) to 0.74 (depression) over approximately a 20 day period. This is consistent with this particular instrument as a measure of mood states, which are expected to vary over time, and supports its construct validity.

### Secondary outcome measures

#### Calgary Symptoms of Stress Inventory (C-SOSI)

This measure is a 56-item scale and is a recent revision of the 95-item Symptom of Stress Inventory (SOSI) [42]. The original SOSI and the C-SOSI are both scales that are designed to measure multiple domains of symptoms of stress, including psychological and physical expressions of stress. The C-SOSI is the product of exploratory factor analysis on SOSI assessment data collected from individuals diagnosed with cancer who attended the Tom Baker Cancer Centre’s MBSR program [42]. A five-point scale (“never” to “very frequently”) is used to rate items based on frequency of stress-related symptoms during a specified time frame selected by the researcher (i.e., the past week). The C-SOSI items form 8 subscales: 1) Depression, 2) Anger, 3) Muscle Tension, 4) Cardiopulmonary Arousal, 5) Sympathetic Arousal, 6) Neurological/GI, 7) Cognitive Disorganization, and 8) Upper Respiratory symptoms. High internal consistency (0.80 to 0.95) has been demonstrated for both the total and subscale scores, as well as good convergent and divergent validity with other well-validated measures [40]. The original SOSI instrument has been used to measure change in symptoms of stress associated with MBSR participation in cancer populations [3,26,43].

#### Post-Traumatic Growth Inventory (PTGI)

This self-report scale is a 21-item inventory that measures the individual’s subjective perception of positive changes following adversity [44]. Individuals are asked to record, on a scale of 0 (not at all) to 6 (very great degree), the level to which their perspective changed as a result of their adversity. Reliability was 0.90 for the normative sample and 0.95 in a sample of individuals with cancer. The test–retest reliability that was measured in the normative sample 2 months later was 0.71.

#### Functional Assessment of Chronic Illness Therapy-Spiritual Well-Being (FACIT-Sp)

This scale is designed to measure spirituality in people with life threatening or chronic illnesses, and includes 12 questions that provide two subscales scores and an overall measure of spirituality [43]. The two subscales (meaning/peace and faith) correspond to one’s sense of meaning and/or purpose in life (e.g., My life lacks meaning and purpose) and one’s comfort and support from their personal faith (e.g., I receive support from my faith). The FACIT-Sp has been established as valid and reliable in individuals with cancer and HIV [44]. Cronbach’s alpha was 0.87 for the overall of spirituality, 0.88 for the faith subscale and 0.81 for the meaning/peace subscale [43].

#### Five Facet Mindfulness Questionnaire (FFMQ)

Baer and colleagues determined that the combined pool of 112 items from five separate mindfulness measures contain five interpretable facets of mindfulness [45]. The analysis revealed five factors that accounted for 33% of the variance. The five facets included in this measure of mindfulness are: attending to sensations, perceptions, thoughts and feelings; describing experience with words; acting with awareness; non-judging of experience; and non-reactivity to inner experience. The FFMQ showed incremental validity in predicting psychological symptoms and correlated strongly to conceptually related variables [45].

### Adverse events

All participants are requested to record and disclose any adverse events in their homework logs, and to report them to the group instructor or the research coordinators. The group instructor also inquires about the participants’ weekly experiences at each class session. Participants are welcomed and strongly encouraged to contact research staff to discuss any questions or events they consider problematic or issues for them regarding the study.

### Analytic strategy

#### Analysis of power for primary effects

The target sample size for this study was based on achieving adequate power for the secondary analyses (since the primary analyses were proportions based on feasibility). The goal was to have 80% power at a significance level of .05, to test the efficacy of the online MBCR intervention in reducing mood disturbance, compared to the control group. On the basis of observed means and standard deviations in three F2F comparable trials [22,26,46]: the estimated effect sizes for group differences in pre- to post-intervention change on the POMS total mood disturbance score was 0.72, 0.51 and 0.58 respectively. Based on the three trials above, and following the more conservative estimation rule proposed by Dattalo (2008) [47] to use t-test estimations for RM-ANOVA designed studies, 26 participants are required for each group to detect a significant difference between the groups. Taking into account the likelihood of 20% attrition as observed in F2F MBSR trials, a total of 32 participants will be recruited for each group—a total sample size of 64 participants.

### Data analysis

All data analysis will be carried out at the completion of the study using the most up-to-date version of SPSS for Windows. Tests will be performed with a two-sided alternative hypothesis, at a critical significance level of 5%. To ensure the appropriateness of the analysis the distributional normality of the data will be confirmed. Wherever possible, p-values and effects sizes will be reported.

### Demographic variables

Participants will be compared using t-tests or chi-square analyses (as appropriate) on primary demographic and psychological variables at baseline to ensure randomization success.

### Primary objective – feasibility

Feasibility will be assessed at the completion of the study through the following measures: 1) Proportion “interested”: this is difficult to ascertain as our reach is hard to know (i.e. the denominator), but we will use the number of invitation letters sent out through the Alberta Cancer Registry as a proxy denominator. The number of patients who phone in with interest in the study will be the numerator (target: 5%). 2) Proportion “eligible”: the number who meet eligibility criteria, over the number who called in (target: 30% - this number is low primarily due to the distress score greater than or equal to 4/10 inclusion criteria). 3) Proportion “consented”: the number of those who consented to the study over the total number who were eligible (target: 85%). Reasons for non-consent will be captured where possible. 4) Proportion “completed”: the number who completed the online intervention, over the number who consented (Target: 85%). Reasons for non-completion will be captured. The study will be deemed feasible if we are within 5% of each target value. Program satisfaction will be assessed through an evaluation form created for the study which assesses participants’ experiences of the MBCR program, the online group, and with the technology.

### Secondary objective – primary outcome

Total Mood Disturbance scores will be examined with a 2 (group) × 2 (time) RM-ANOVA to examine main effects of time and group, and any interactions between time and group. Post-hoc analyses will be conducted to examine the simple main effects for treatment if a significant interaction is detected.

### Secondary objectives

Secondary Outcomes: The same analytic strategy will be applied to the secondary outcomes of symptoms of stress, mindfulness, spirituality and posttraumatic growth.

## Discussion

Recruitment for this trial has proceeded as anticipated; with participants arriving through the Alberta Cancer Registry via study invitation letters, through rural cancer centre posters, pamphlets, advertisements, and community-based networks. To date, 1800 individuals have been contacted through the Alberta Cancer Registry through mailed study invitation with 157 responding. Of those, 41 were ineligible (8 deceased, 3 outside of primary treatment inclusion criteria, 12 had scheduling conflicts, 9 had low distress, 6 had no computer/high speed Internet, 2 were living outside Alberta, and one individual was not diagnosed with cancer). 54 declined participation (with the majority stating they were not interested in the intervention, while 10 stated they were effectively managing stress through other methods. 5 asked to be contacted later or considered for the program at a later date. 57 participants have been enrolled thus far in the program: 2 withdrew (1 MBCR, 1 wait-list). Reasons for discontinuing with the study included treatment related issues and schedule change. 47 have completed the program or in the process of completing the study. No adverse events have been reported. Final analysis of the data is not yet available to compare the intervention and control groups; however, several preliminary comments and conclusions can be made from the experience of the trial to-date.

### Recruitment challenges

While recruitment began slower than our initial anticipated timeline for the trial, recruitment through the Alberta Cancer Registry has resulted in enrolling individuals diagnosed with cancer who are distressed and interested in participating in an online MBCR program, and who would otherwise be unable to access such a program. Recruitment will plan to continue through April 2013, with full analysis of data to follow.

Limited or slow Internet access or computer-illiteracy has restricted some participants enrolling in the program; however, statistics in Alberta for rural Internet connectivity and the rising rates of computer use are encouraging, and multiple avenues of recruitment have provided successful recruitment of eligible participants to date.

The online MBCR intervention, as with F2F MBSR programs require participants to be willing to commit to attending 8-weekly 2-hour Wednesday evening classes and a Saturday full day retreat. Many individuals interested in the program have busy schedules, especially the individuals currently receiving cancer treatments, and the program is a significant time commitment. Nevertheless, significant research team efforts have allowed for successful recruitment strategies. Initial response to the intervention from participants has been positive.

### Study benefits

Online MBSR interventions offer promise for improving the accessibility of evidence-based psychosocial interventions for underserved individuals. Synchronous online therapy is available to anyone with Internet access. By allowing individuals diagnosed with cancer to participate in group interventions without leaving their homes, common reasons for not attending or prematurely discontinuing F2F groups may be reduced, including long travel distances and issues with mobility (which may include driving issues, busy roads, parking, walking etc.).

F2F MBSR programs reduce mood disturbance, decrease symptoms of stress, and improve quality of life in individuals diagnosed with cancer, but due to geographical and illness-related barriers, it is often inaccessible. This trial is incorporating sophisticated real-time technology to reach underserved individuals diagnosed with cancer who are currently often excluded from MBSR programs, with the goal of improving access to psychosocial interventions for a difficult to reach population.

Online MBSR programs led by experienced instructors have the potential to disseminate psychosocial interventions to underserved individuals, regardless of both participant and instructor geographical location. Despite the demonstrated effectiveness of F2F MBSR, and ease of use of available technology, the effectiveness of an online MBSR program for individuals diagnosed with cancer has yet to be evaluated. Such a program could potentially improve access to evidence-based psychosocial programs in Alberta and beyond. The results of this study may help propel further research in the integration of mind-body medicine and technology for underserved populations.

## Competing interests

Kelley McCabe is the CEO and Founder of eMindful. Kelley McCabe also has an investment in eMindful. Kelley McCabe will provide in-kind support for the facilitation of the online MBCR programs through eMindful, but has played only a technical role in study design and will not be involved in data analysis or interpretation. Steven Flowers is an employee of Mindful Living Programs. Steven Flowers has been involved in the implementation of this trial by facilitating the online mindfulness classes, and will help with interpretation by editing manuscripts, but has not, or will not have involvement in the funding of this trial or data analysis.

## Authors’ contributions

All authors participated in the design of the study and development of research protocols, as well as contributing to and approval of the final manuscript. All authors read and approved the final manuscript.

## Pre-publication history

The pre-publication history for this paper can be accessed here:

http://www.biomedcentral.com/1472-6882/13/34/prepub
